# Pulmonary Tuberculosis due to *Mycobacterium bovis* in Captive Siberian Tiger

**DOI:** 10.3201/eid0911.030297

**Published:** 2003-11

**Authors:** Ákos Lantos, Stefan Niemann, László Mezősi, Endre Sós, Károly Erdélyi, Sándor Dávid, Linda M. Parsons, Tanja Kubica, Sabine Rüsch-Gerdes, Ákos Somoskövi

**Affiliations:** * Semmelweis University, Budapest, Hungary; † National Reference Center for Mycobacteria, Borstel, Germany; ‡ Budapest Zoological and Botanical Garden, Budapest, Hungary; § Central Veterinary Institute, Budapest, Hungary; ¶ Korányi National Institute for Tuberculosis and Respiratory Medicine, Budapest, Hungary; # New York State Department of Health, Albany, New York, USA; ** University at Albany, Albany, New York, USA

## Abstract

We report the first case of pulmonary tuberculosis caused by *Mycobacterium bovis* subsp. *caprae* in a captive Siberian tiger, an endangered feline. The pathogen was isolated from a tracheal aspirate obtained by bronchoscopy. This procedure provided a reliable in vivo diagnostic method in conjunction with conventional and molecular tests for the detection of mycobacteria.

*Mycobacterium bovis*, a member of the *M. tuberculosis* complex *(*MTBC), can cause tuberculosis in a wide range of domestic and wild animals and also in humans (*1*,*2*). Routine differentiation of *M. bovis* is based on a number of phenotypic characteristics and biochemical tests (*2*). *M. bovis* shows dysgonic growth on Löwenstein-Jensen (LJ) medium and has been described as negative for nitrate reduction and niacin accumulation (*2*). As a further criterion for the differentiation of *M. bovis*, intrinsic resistance to pyrazinamide (PZA) has been described (*2*). However, more recently, PZA-susceptible strains of *M. bovis* were found in Spain and Germany; these strains were also characterized by specific molecular techniques (*3*–*5*). As a consequence, *M. bovis* was split into two subspecies: *M. bovis* subsp. *bovis*, which showed resistance to PZA, and *M. bovis* subsp. *caprae*, which was sensitive to PZA (*6*,*7*). *M. bovis* subsp. *caprae* was initially isolated from sheep and goats in Spain (*3*,*4*,*7*); however, further studies confirm its infectivity in humans, cattle, and red deer (*6*,*8*). We report the unusual case of a *M. bovis* subsp. *caprae* infection in a captive Siberian tiger.

## Case Report

**Figure 1:**
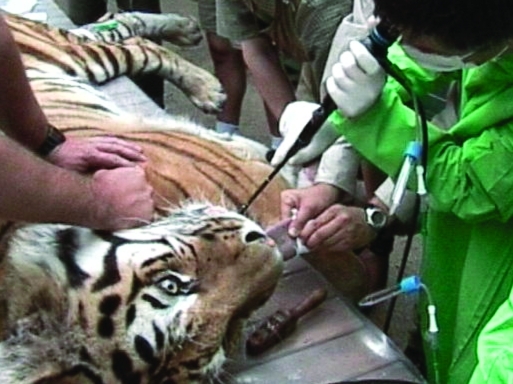
Obtaining a tracheal washing of the Siberian tiger by bronchoscopy.

An 8-year-old male Siberian tiger at the Budapest Zoological and Botanical Garden had episodes of coughing in October 2001. Because the coughing did not stop in 6 to 7 days, an expectorant (Bisolvon; Boehringer Ingelheim Vetmed Gmbh., Ingelheim am Rhein, Germany) was given for 10 days. His condition showed a temporary improvement; however, after a few weeks, the animal started coughing again, and his appetite decreased. Amoxicillin plus clavulanic acid (Amoksiklav; Lek Animal Health, Ljubljana, Slovenia) and ketoprophen (Ketofen, Merial, Lyons, France) therapy was given for 7 days. The tiger’s condition did not show any notable improvement. In addition, in May 2002, the animal’s respiratory rate became elevated, he became dyspneic and emaciated, and his daily activity substantially decreased. Further antibacterial treatment was administered (cefatroxil, Cefa-cure; Intervet, Boxmeer, the Netherlands) during that month without clinical effect. At that point, the animal was anesthetized, and tracheoscopy was performed with a flexible 56-cm bronchoscope (Olympus B3R; Tokyo, Japan (Figure 1). The examination found a large amount of purulent mucus in the trachea. Therefore, several tracheal washings were taken for microbiologic tests by using a commercially available tracheal suction set (Medinorm Medizintechnik GmbH, Quierschied, Germany (Figure 1). A chest radiograph showed a severe and extensive bronchointerstitial pattern with cavernous lesions in both lungs.

Nine days after the specimens were taken, cultures for mycobacteria showed growth in the broth-based MGIT 960 system (Becton-Dickinson Microbiology Systems, Sparks, MD). The acid-fast organism that was isolated was identified as MTBC by the AccuProbe TB assay (Gen-Probe Inc., San Diego, CA).

Since the tiger had stopped eating and his condition had dramatically deteriorated, the animal was euthanized and necropsy was performed. Hematoxylin and eosin–stained histologic sections of the lung segments showed an extensive multifocal infiltration of lymphocytes, histiocytes, and some scattered multinuclear giant cells within the framework of proliferated connective tissue and collagen fibers of the cavernous lesions. Ziehl-Neelsen staining showed an intracellular accumulation of acid-fast bacteria in several alveolar macrophages and epithelioid cells.

The keepers of the tiger also underwent pulmonary radiographs and tuberculin skin testing. Their skin test results were negative, and clinical or radiologic signs of tuberculosis were not detected.

### Characterization of MTBC Isolate

Colony morphology of the isolated MTBC strain showed dysgonic growth on LJ medium and microaerophilic growth on Lebek medium. The strain was susceptible to PZA (100 μg/mL) and thiophen-2-carboxylic acid hydrazide (TCH; 1 μg/mL) and negative for niacin accumulation and nitrate reduction (*9*–*11*).

**Figure 2:**
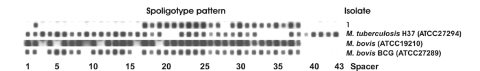
Spoligotype patterns of the isolate obtained from the Siberian tiger: lane 1; spacer sequences 3–16 and 39–43 are absent); lane 2, control strain *Mycobacterium tuberculosis* H37Rv ATCC 27294; lane 3, control strain *M. bovis* ATCC 19210; lane 4, *M. bovis* BCG ATCC 27289.

The genome of the isolate was analyzed for specific mutations in the *pncA*, *oxyR*, and *gyrB* genes by automated DNA sequencing, polymerase chain reaction–restriction fragment length polymorphism (PCR-RFLP) technique, and spoligotyping as described previously (*5*,*12*–*16*). Susceptibility of the isolate to PZA was linked with a wild-type *pncA* sequence. In addition, the isolate contained the *M. bovis*–specific G-to-A mutation at position 285 in the *oxyR* gene and the G-to-A mutation at position 756 in the *gyrB* gene. However, spoligotyping showed a pattern with the absence of spacer sequences 39–43 and 3–16 (Figure 2), and T to G mutation at position 1,311 in the *gyrB* gene, characteristic of *M. bovis* subsp. *caprae,* could also be detected by DNA sequencing. On the basis of these phenotypic and genetic characteristics, the strain was identified as *M. bovis* subsp. *caprae* (*3*,*6*,*7*,*17*).

## Conclusions

MTBC comprises these closely related organisms: *M. tuberculosis;* *M. africanum; M. bovis*; the vaccine strain, *M. bovis* bacillus Calmette-Guérin; and three rarely seen members, *M. microti*, *M. canettii,* and the recently described seal bacillus, *M. pinnipedii* (*17*–*20*). Differentiation within MTBC is necessary for individual patient treatment (i.e., inclusion or exclusion of PZA) and for epidemiologic purposes, especially in areas of the world where tuberculosis has reached epidemic proportions or wherever the transmission of *M. bovis* between animals, animal products, and humans is a problem (*10*).

The host range of *M. bovis* is wide, including many animal species and humans. Carnivores such as large felines may acquire the infection through the alimentary tract by eating infected meat (*4*). Reports of tuberculosis in large captive or free-living felines are not common (*21*–*26*), however.

To our knowledge, this case is the first in which tuberculosis attributable to *M. bovis* subsp. *caprae* was diagnosed in a large feline. The rapid and accurate in vivo diagnosis of tuberculosis is indispensable in endangered captive animals such as the Siberian tiger, not only because of the declining population of this species but also to prevent the transmission of the disease to other animals. Although nasal or throat swabs are used most often, we found tracheal washing by bronchoscopy was easy to perform, rapid, and more adequate than swabs (provided a larger sample volume from the lower airways) for obtaining clinical specimen for mycobacterial or other microbiologic tests.

The rapid diagnosis of tuberculosis is essential for adequate antituberculosis treatment to be started as early as possible. The effectiveness of antituberculosis therapy in felines is controversial (*27*). However, when an endangered animal is involved, early diagnosis of the disease might help control it in time to save the animal, especially with the help of a rapid in vivo diagnostic method such as tracheal washing through bronchoscopy. Tracheal washing can also be the method of choice to bacteriologically monitor the efficacy of therapy. In this case, the poor appetite and condition of the animal did not allow survival long enough for delivery of antituberculosis treatment. The source of infection could not be conclusively identified retrospectively; infected goat meat (a usual diet of the animal) is a likely possibility because the tuberculosis-related control measures are not as strict with goats as with cattle in Hungary (annual tuberculin skin testing of goats is not mandatory, for example) (*28*).

This report indicates that routine differentiation within the MTBC is indispensable for understanding the epidemiology of tuberculosis and for determining the prevalence, transmission, and clinical importance of the different members of the complex.
